# Supplementary material for: Safety and Immunogenicity of the Intranasal Vaccine Candidate Mambisa and the Intramuscular Vaccine Abdala Used as Booster Doses for COVID-19 Convalescents: A Randomized Phase 1–2 Clinical Trial
Source: Vaccines (Basel). 2024 Sep 1;12(9):1001. doi: 10.3390/vaccines12091001 (PMC11435458; doi:10.3390/vaccines12091001)
Supplement: Supplementary file 1 [file vaccines-12-01001-s001.zip › vaccines-3074328-supplementary.pdf]

SUPPLEMENTARY MATERIALS

Table of Contents

- Abdala-Mambisa Clinical Trial Research Group 2
  1. National Coordinating Center for Clinical Trials (CENCEC) 2
  2. Clinical sites 2
     a. Hermanos Ameijeiras Clinical-Surgical Hospital, La Habana (Main study execution site) 2
     b. Manuel Ascunce Domenech Provincial Clinical-Surgical Teaching Hospital, Camagüey 3
     c. Pedro Raúl Sánchez Teaching Polyclinic, Pinar del Río 4
     d. Saturnino Lora Provincial Clinical-Surgical Hospital, Santiago de Cuba 4
  3. Subjects referral and recruitment 5
- Authors name and surname, email and ORCID 6
- Nasal devices for Mambisa vaccine delivery: Specifications, use instructions and images 7
  1. Intranasal atomization device- Device AZ (CNEURO, Cuba) 7
  2. Nasal spray-Device S (GAASCH PACKAGING Ltd., UK) 8
  3. Nasal dropper-Device D (Sopac Medical, France) 9
  4. Intranasal atomization device-Device AZ (Wuxi NEST Biotechnology Co. Ltd, China) 10
- Exclusion criteria for study participants. 12
- Quantification of IgA antibodies using an in-house ELISA test (CIGB, Cuba). 13

- **Abdala-Mambisa Clinical Trial Research Group**

1. **National Coordinating Center for Clinical Trials (CENCEC)**

Gladys Jiménez-Rivero. Data Management Manager
Isabel López-Zayas. Randomization Manager
Miladys Reyes-Ortiz. Randomization Manager
Dyleye Estrada-Pérez. Randomization Manager
Olga María Padrón-Labrador. Medical Supplies Manager
Dámaris Méndez-Rueda. Clinical Trial Monitoring
Yulka Prieto-Ferro. Responsible for Conducting and Monitoring the Clinical Trial in Pinar del Río Province.
Yoryana Ramírez-Sánchez. Responsible for Conducting and Monitoring the Clinical Trial in Pinar del Río Province.
Mayler Ramírez-Sosa. Responsible for Conducting and Monitoring the Clinical Trial in Camagüey Province.
Aymara Margenat-Pérez. Responsible for Conducting and Monitoring the Clinical Trial in Camagüey Province.
Sanlia Landasuri-Llago. Responsible for Conducting and Monitoring the Clinical Trial in Santiago de Cuba Province.
Dianne Yurien Griñan-Semaná. Responsible for Conducting and Monitoring the Clinical Trial in Camagüey Province.
Alicia Rodríguez-Bernabé. Responsible for Conducting and Monitoring the Clinical Trial in Las Tunas Province.
Dr. Iralys M Benítez-Guzmán. Responsible for Conducting and Monitoring the Clinical Trial in Sancti Spiritus Province.
Dr. Héctor S Ruiz-Calabuch. Responsible for Conducting and Monitoring the Clinical Trial in Sancti Spiritus Province.

2. **Clinical sites**

   a. **Hermanos Ameijeiras Clinical-Surgical Hospital, La Habana (Main study execution site)**

Dr. Emilio Fidel Buchaca-Faxas. Investigator
Dr. Lais Rodríguez-Amador. Investigator
Dr. Juan Miguel Baiz-López. Investigator
Dr. Eglis Ceballos-Rodríguez. Investigator
BSc. Sergio Guido Llinás-Carrillo. Clinical Trials Coordinator
BSc. Nirka López-León. Clinical Research Coordinator
BSc. Yohanka Avila-Padrón. Nursing
Dr. Marcia L. Hart-Casarez. Co-Investigator
BSc. Ada Lidia López-Suarez. Co-Investigator
BSc. Wendy Caraballo-Rodríguez. Co-Investigator
BSc. Biler Salcedo-González. Co-Investigator
Tech. Reyna Raysa-Medina. Co-Investigator
Dr. Miroslaba Dalas-Guiber. Co-Investigator
BSc. María Elena Suardiaz-Espinosa. Co-Investigator
BSc. Ana María Arias-Prieto. Co-Investigator
BSc. Regla Ramos-Duarte. Co-Investigator
Tech. Greisy Feijoo-Martín. Co-Investigator

BSc. Grisell González-Mir. Co-Investigator
BSc. Ivelisse N. Fleites-León. Co-Investigator
Tech. Arletys Molina-González. Co-Investigator
Dr. María Amparo Navarro-Fernández. Co-Investigator
BSc. Gilda Machado-González. Co-Investigator
Dr. Alexis Morales-Villalba. Co-Investigator
Dr. Yoisis Noa-Oms. Co-Investigator
Dr. Soleil Morales-Díaz. Co-Investigator
Dr. Idalberto Vega-Bayard. Co-Investigator
Dr. Genma Salas-Cruz. Co-Investigator
Dr. Yomayra C. Ocampo-Carrión. Co-Investigator
Dr. Sergio J. Sánchez-Hernández. Co-Investigator
Dr. Damián Torres-Santulland. Co-Investigator
BSc. Naibel García-Padrón. Co-Investigator
Dr. Osman Perdomo-Verdecia. Co-Investigator
Dr. Mayla Cordiez-Pérez. Co-Investigator
BSc. Juana L. Andux-Valdés. Co-Investigator
BSc. Greisy Feijoo-Martín. Co-Investigator
Dr. Yaimé Bencomo-Alamo. Co-Investigator
Dr. Liem Estrella Hernández-Cuesta. Co-Investigator
Dr. Yudith Peña-Garcell. Co-Investigator
Dr. Liudmila Hernández-Cardoso. Co-Investigator
BSc. Diamela Monteagudo-Mugara. Co-Investigator

b. Manuel Ascunce Domenech Provincial Clinical-Surgical Teaching Hospital, Camagüey

Dr. Yanara Nélida Guerra-Peláez. Investigator
Dr. Olga Georgina Caveda-Estela. Investigator
Dr. Alina Tejeda-Fuentes. Investigator
Dr. Iliana Mesa-Pedroso. Investigator
Dr. Gloria García-González. Investigator
BSc. Idalmis Rivera-Blas. Co-Investigator
BSc. Alexis Torrens-Llanes. Co-Investigator
BSc. Yudenia Machado-Noa. Co-Investigator
BSc. Mayelin Ortega-Kirkort. Co-Investigator
BSc. Yadira Machado-Castro. Co-Investigator
BSc. Yordanis García-Godinez. Co-Investigator
BSc. Marbelis Pereira-Otero. Co-Investigator
BSc. Judith Ojeda de Pedro. Clinical Research Coordinator
BSc. Yaneydis Lores-Méndez. Clinical Research Coordinator
Dr. Belquis Hernández-García. Investigator
Dr. Yasinleidys Malo-Lantigua. Investigator
Dr. Ilina Mursuli-García. Investigator
Dr. Osmara Silvia-Cabrera Hernández. Investigator
Dr. Evelin Mariam-Roberts Dandie. Investigator
BSc. Lidia López. Investigator
Dr. Marcia Noy-León. Investigator
BSc. Ana Iris Orasma-Peña. Investigator
Dr. Kenia Montenegro-Guerra. Investigator

Dr. Nilda Alemañy-Bueno. Investigator
Dr. Mercedes Milagros Varona-Arias. Investigator
BSc. Dayetsi Parrado-Cardoso. Co-Investigator
Dr. Ivania Álvarez-Silveira. Co-Investigator

c. Pedro Raúl Sánchez Teaching Polyclinic, Pinar del Río

Dr. Dagneris de la Cruz-Pérez. Investigator
Dr. Gilberto Mujica-Madera. Investigator
BSc. Dianelis Ovalle-Díaz. Investigator
BSc. Luis Carrillo-Blanco. Investigator
BSc. Yaquelín Boligan-Carrillo. Investigator
BSc. Maida Fleita-Rodríguez. Investigator
BSc. Rosario Carrodegua-Mijares. Co-Investigator
BSc. María de las N. Pérez-Rodríguez. Co-Investigator
BSc. María L. Rodríguez-Rivera. Co-Investigator

d. Saturnino Lora Provincial Clinical-Surgical Hospital, Santiago de Cuba

Dr. Eduardo Alejandro Ortiz-Artigas. Co-Investigator
Dr. Lianne Del Toro-la Hera. Co-Investigator
BSc. Yaquelin Naranjo-Vargas. Clinical Research Coordinator
BSc. Ahimara Rosado-Rosado. Clinical Research Coordinator
Dr. Dayami Zayas-Ordoñez. Co-Investigator
Dr. Yuleidis Scamith-Bosa. Co-Investigator
Dr. Raico De La Torres-Domínguez. Co-Investigator
Dr. Sergio Del Valle-Piñera. Co-Investigator
Dr. Yanelatcy Pozo-Despaigne. Co-Investigator
Dr. Conrado Enrique Hernández-Pérez. Co-Investigator
MSc. María Del Carmen Clares-Pochet. Co-Investigator
Dr. Eddy Williams-Corona. Co-Investigator
BSc. Elizabeth Ramos-Caraballo. Co-Investigator
BSc. Ledis Nancy Garbey-Delas. Co-Investigator
BSc. Juliet Sigüenza-Castilla. Co-Investigator
BSc. Yurina Álvarez-Calvo. Co-Investigator
BSc. Virgen Laigen Ruano-González. Co-Investigator
BSc. Odalis Pol-Fis. Co-Investigator
BSc. Luisa Amalis Recacen. Co-Investigator
BSc. Mercedes Yaima Ferrer-Bonne. Co-Investigator
Dr. Alfredo Hernández-Magdariaga. Co-Investigator
PhD. Josefa Bell-Castillo. Co-Investigator
Dr. Yanet Durive-Pérez. Co-Investigator
Dr. Yadira Díaz-Castillo. Co-Investigator
Dr. Ulda Falagán-Vázquez. Co-Investigator

3. Subjects referral and recruitment

Municipal Health Directorate in Central Havana:
Dr. Alfredo Díaz-Ferrer.
Dr. Omar Benito Tamayo-Serrano.
Dr. Maricela Cangas-Crespo.
Dr. Venecia Pereira-García.

Sancti Spiritus Province (Recruitment for the Manuel Ascunce Domenech clinical site in Camagüey):
Dr. Niuvis Fundora-Martín. Provincial Center of Hygiene and Epidemiology.
PhD. Enrique Pérez-Cruz. Director of Sancti Spiritus CIGB.

- **Authors name and surname, email and ORCID**

NAME AND SURNAME	Email	ORCID
MSc. Gilda Lemos-Pérez	gilda.lemos@cigb.edu.cu	https://orcid.org/0000-0003-0596-6501
MSc. Yinet Barrese-Pérez	yinet@cencec.sld.cu	
Ing. Yahima Chacón-Quintero	yahima.chacon@cigb.edu.cu	
PhD. Rolando Uranga-Piña	rolando@cencec.sld.cu	
MSc. Yisel Avila-Albuerne	yisel@cencec.sld.cu	
Dr. Iglermis Figueroa-García	iglermis@infomed.sld.cu	
Dr. Osaida Calderín-Marín	cmosaida.cmw@infomed.sld.cu	
Dr. Martha M. Gómez-Vázquez	dirpolrs@princesa.pri.sld.cu	
Dr. Marjoris Piñera-Martínez	marjorisp@infomed.sld	
BSc. Sheila Chávez-Valdés	sheila.chavez@cigb.edu.cu	
BSc. Ricardo Martínez-Rosales	ricardo.martinez@cigb.edu.cu	
BSc. Lismary Ávila-Díaz	lismary.avila@cigb.edu.cu	
Tech. Amalia Vázquez-Arteaga	amalia.vazquez@cigb.edu.cu	
Tech. Hany Lianet González-Formental	hany.gonzalez@cigb.edu.cu	
Tech. Giselle Freyre-Corrales	giselle.freyre@cigb.edu.cu	
Tech. Edelgis Coizeau-Rodríguez	edelgis.coizeau@cigb.edu.cu	
PhD. Miladys Limonta-Fernández	miladys.limonta@cigb.edu.cu	
PhD. Marta Ayala-Avila	marta.ayala@cigb.edu.cu	
PhD. Eduardo Martínez-Díaz	eduardo@citma.gob.cu	
PhD. Eulogio Pimentel-Vazquez	eulogio@oc.biocubafarma.cu	
PhD. Gerardo Guillén-Nieto	gerardo.guillen@cigb.edu.cu	https://orcid.org/0000-0003-3098-0970

- **Nasal devices for Mambisa vaccine delivery: Specifications, use instructions and images**

Phase 1

1. **Intranasal atomization device- Device AZ (CNEURO, Cuba)**

Device specifications

ATOMIZER KIT FOR DRUG ADMINISTRATION-NEURONIC AF-1.0

The Neuronic AF-1.0 Drug Delivery Atomizer Kit is intended to convert liquids into very fine atomized particles for rapid tissue absorption. Its operating principle allows the exact dose of drugs to be administered in any position. It is a non-invasive and easy-to-use route of administration.

The Neuronic AF-1.0 Drug Delivery Atomizer Kit is indicated to administer 2 doses of 0.1 mL of drug via the nose or on the surface of tissues of the human body according to medical indication, thus having a greater effect on the patient. It is designed for single use. The device components are showed in Figure S1.

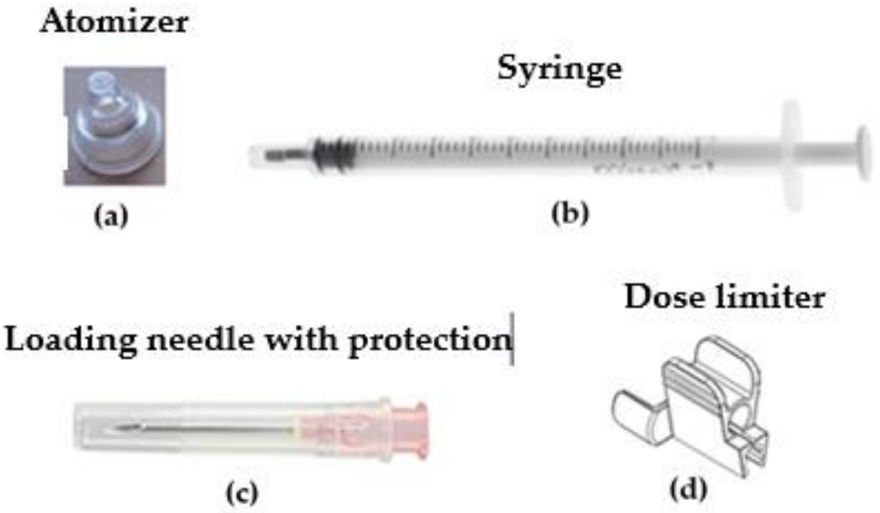

Figure S1. Components of the intranasal atomization device (Device AZ). (a) Medical grade transparent plastic material atomizer; (b) 1 mL capacity syringe, Luer-Slip connection type, made of transparent plastic material; (c) Loading needle: Hypodermic needle with safety protection. Diameter 18G (1.2 mm) and length 1½ inches (40 mm) and (d) Dose limiter: Opaque white plastic material. Its design allows it to be easily attached to the syringe. It is calibrated so that, with the syringe provided in the kit, it can administer the exact dose of drug according to the medical indication.

Procedure for using Device AZ (CNEURO, Cuba):

Step 1: Load the syringe with approximately 0.25 ml of vaccine and press the push button to 0.2 ml to remove air.

Step2: Mount the dose limiter in the push rob, remove the needle and place the atomizer.

Step 3. Gently aim the spray nozzle on one nasal cavity and quickly press the push rob until the top of dose limiter meet the bottom of the tube. The 0.1 mL dose injection was completed.

Step 4. Remove the dose limiter and gently aim the spray nozzle in another nasal cavity and quickly press the push rob until the bottom.

2. Nasal spray-Device S (GAASCH PACKAGING Ltd., UK)

Device specifications

Name: SP2V NASAL SPRAY DIN 18 +. Code: 790035AB.

- It has a mechanism that prevents the flow of return air, thus ensuring that there is no contamination inside.
- The system and structure are safe; it has no risk of contamination by external microorganisms, neither at rest nor during operation.
- It contains a 0.2 μm filter membrane in the ventilation system to avoid contamination of the product through filtration of microorganisms.
- There are no anti-microbial additives within the pump components.
- The fluid path is free of metals.
- It has anti-clogging technology at the tip of the seal, preventing the crystallization of viscous and volatile formulations.
- Risk Class I Nasal Device / Sterile by Gamma Irradiation with Cobalt-60 (60Co).
- Includes security seal.
- Fits into glass bottles of 5 to 20 mL.
- Good pre-compression for reproducible atomization execution between 100 and 140 μL.

The device components are showed in Figure S2. More information available at https://catalogue.gaaschpack.eu/catalog/i/pharma/accessoires/dispensers/790035ab/sp2v-nasal-spray-din-18--

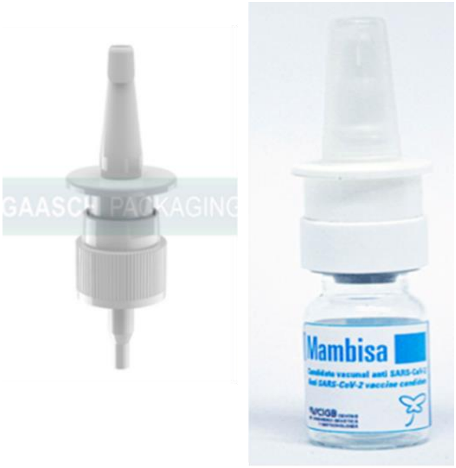

(a) (b)

Figure S2. Components of the nasal spray device (Device S). (a) The nasal spray component; (b) the nasal spray component attached to the Mambisa vaccine.

Procedure for using Device S (see Figure S3):

Step 1. Remove the plastic seal from the bulb.
Step 2. Place the safety seal on the nasal device.
Step 3. Place the nasal device on in the mouth of the vial in a slightly inclined tilted position and press down until it is subject fixed by the clip-on closing system.
Step 4. Remove the security seal.
Step 5. Partially withdraw the protective cap and press twice, so that the medication stays in its interior (This procedure avoids dispersing the aerosol into the air and fills the internal chamber of the device).
Step 6. Set the subject with the head slightly tilted back with support throughout the procedure. Apply the product into the first nostril after exhaling, keeping maintaining the head position for one minute; repeat the same procedure is repeated in the other nostril.

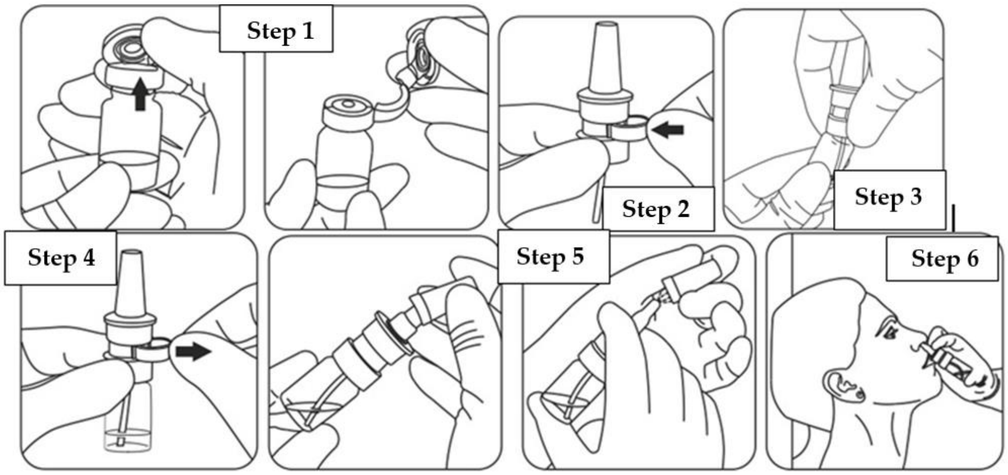

Figure S3. Steps for nasal spray device (Device S) preparation and nasal application.

3. **Nasal dropper-Device D (Sopac Medical, France)**

Device specifications

20 mm non-pigmented natural polyethylene dropper cap, 0.85 mm thick (sterilized by gamma radiation) with a protector on the tip of the dropper, with a sterilization indicator that can be found located on the primary container in contact with the product (4 droppers in 1 blister), in the multipack that contains several blister packs with dropper caps or in the corrugated cardboard box that contains several dropper caps. The sterilization date and expiration date of the droppers are found on the primary packaging.

Available for 13 mm/20 mm ISO 8368 necks. The device components are showed in Figure S4. More information available at https://www.sopac-medical.com/dropper-to-crimp-for-13-mm-and-20-mm-necks/

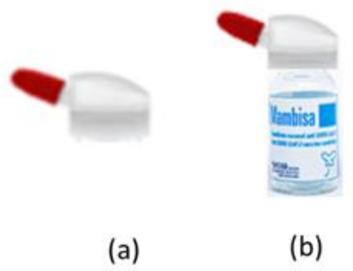

Figure S4. Components of the nasal dropper (Device D). (a) Dropper to crimp with the red cap; (b) (b) The dropper attached to the Mambisa vaccine.

Procedure for using Device D:

Step 1. Carefully remove the plastic seal from the bulb.
Step 2. Place the snap cap on the neck of the vaccine vial, pressing until it fits firmly.
Step 3. Position the subject with the head slightly tilted back and supported throughout the procedure.
Step 4. Remove the red cap. Invert the dropper and press the dropper cap, applying two drops to the first nostril after exhaling, maintaining the head position for one minute. Repeat the same procedure is repeated in the other nostril.

Phase 2

4. **Intranasal atomization device-Device AZ (Wuxi NEST Biotechnology Co. Ltd, China)**

Device specifications

Disposable intranasal atomization device. Cat. No. 201002

Product presentation:

• The device provides a rapidly absorbed and painless medication delivery option for non-invasive intranasal medication delivery. It improves safety for both caregivers and patients by preventing needle-stick injuries.

• It is an atomizing drug delivery device intended to convert liquid preparations into atomized particles and spray onto the surface of tissues (or organs) of the human body for full contact to maximize the delivery effect. Meanwhile, the reasonable and effective self-destructive structure ensures that the product can only be used once to provide users with safe and hygienic products.

Characteristics:

1. Rapid absorption: Since since a specially designed nasal spray device could translate convert o transform the liquid into atomized particles, this device will promote the rapid absorption of the drug.
2. Non-invasive and painless: The umbrella-shaped spray device will not cause any damage or irritation to the human body.
3. Self-destruct device: Self-destruct device design ensures disposable use.3. Self-destruct device: Self-destruct device design ensures disposable use.
4. Special dose limiter: easy to mount and remove thanks to the special design of the dose limiter as a clamp.

5. Clear scale
6. Inoculate with precision: Using a dose limiter, it is possible to inoculate with precision.

The device components are showed in Figure S5.

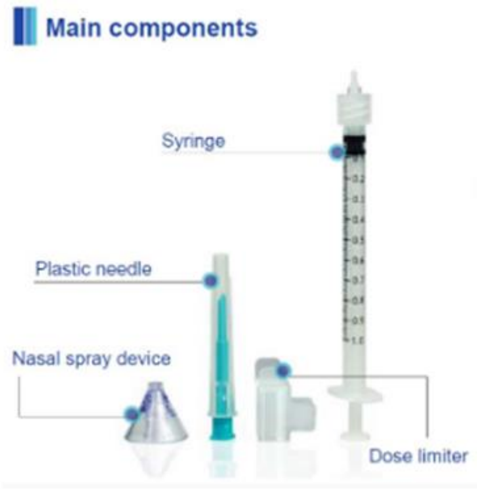

Figure S5. Components of the intranasal atomization device-Device AZ (Wuxi NEST Biotechnology Co. Ltd, China).

Procedure for using Device AZ (Wuxi NEST Biotechnology Co. Ltd, China):

Step 1: Load the syringe with approximately 0.3 ml of vaccine.
Step2: Remove the needle and screw on the atomizer.
The rest of the procedure is detailed in Figure S6

More information available at https://cell-nest.ir/products/disposable-intranasal-atomization-device/disposable-intranasal-atomization-device.pdf. Also, a video is available at https://www.youtube.com/watch?v=H5tnqmnhGGA

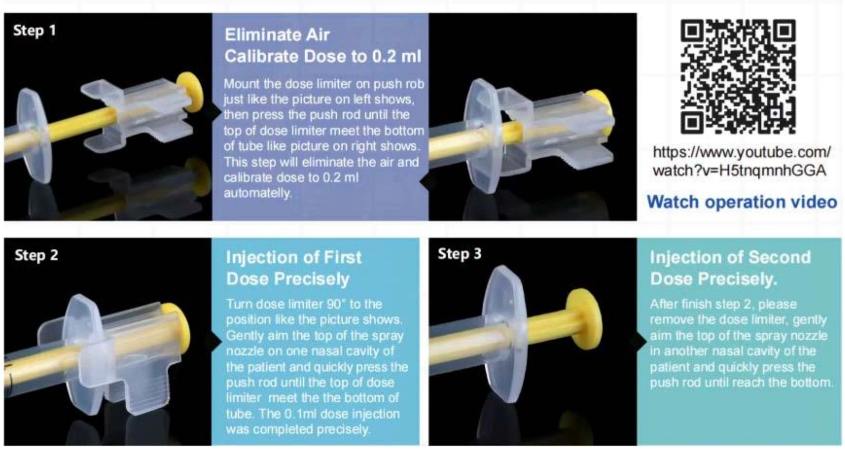

Figure S6. Steps for intranasal atomization device AZ preparation and nasal application.

- **Exclusion criteria for study participants.**

Acute SARS-CoV-2 infection in the last two months, contact or suspect of COVID-19 cases, symptoms similar to COVID-19 in the previous 15 days (fever, cough or shortness of breath, loss of smell or anosmia, loss of taste or ageusia, and any acute infection 15 days prior to inclusion). Individuals with decompensated chronic diseases. Finding or medical condition in the nasal passages that makes difficult proper vaccine administration and monitoring (e.g. chronic obstructive allergic rhinitis, obstructive deviation of the nasal septum, or benign and malignant tumors of the nasal cavity such as polyposis and squamous cell carcinoma). Subjects with tattoos in both deltoid regions. Body mass index (BMI) ≤18 or ≥35 kg/m2. People who received treatment with a specific coronavirus vaccine (authorized or investigational), or any investigational product three months prior to enrollment or are scheduled for treatment during the study. Subjects with any medical condition requiring an immunomodulator (steroid or cytostatic). People who received treatment with blood or any blood product within three months prior to inclusion. Other reasons for exclusion were alcohol or drug abuse, pregnancy or desire to become pregnant, breastfeeding, and psychiatric or mental disorders.

- **Quantification of IgA antibodies using an in-house ELISA test (CIGB, Cuba).**

High binding 96-wells polystyrene plates (Costar, USA) were coated with monomeric RBD-His protein (Molecular Immunology Center, CIM, Cuba) at 500 ng per well in 0.1 M sodium carbonate-bicarbonate buffer pH 9.6 for 1 hour. After a wash step with 0.1% (v/v) Tween-20 in distilled water, plates were blocked with 2.5% (w/v) skimmed milk in phosphate buffered saline (PBS) and 0.05% (v/v) Tween 20 for 1 hour. After a wash step, samples, the reference material and assay controls were added at proper dilutions and incubated for 1 hour. The reference material (CoVIg-03-0222), consisting in a pool of high anti-RBD IgA titer sera, was employed for the calibration curve (dynamic range 1.4–185 AU/mL). After four wash steps, a goat anti-human IgA-peroxidase conjugate (Sigma, USA) was added and incubated for 30 minutes. All incubations were carried out at 37 °C. After a washing step, a TMB substrate solution in citrate buffer (pH 5.0) with 0.015% hydrogen peroxide was added to the plates and incubated for 10 minutes in the dark at 22–25 °C. An automated ELISA reader (Tecnosuma, Cuba) recorded the absorbance at 450 nm. Titers were given in AU/mL with a detection limit of 0.6.
